# Combined Hurdle Technologies Using UVC Waterproof LED for Inactivating Foodborne Pathogens on Fresh-Cut Fruits

**DOI:** 10.3390/foods10081712

**Published:** 2021-07-23

**Authors:** Geun-Hyang Kim, Chae-Lim Lee, Ki-Sun Yoon

**Affiliations:** Department of Food and Nutrition, College of Human Ecology, Kyung Hee University, 26 Kyungheedae-ro, Dongdaemun-gu, Seoul 02447, Korea; dytnfvos@naver.com (G.-H.K.); crl95@khu.ac.kr (C.-L.L.)

**Keywords:** fresh-cut fruits, slightly acidic electrolyzed water (SAEW), fumaric acid (FA), ultravioletC waterproof light-emitting diodes (UVC W-LED), hurdle technology

## Abstract

This study investigated the combined bactericidal efficacy of slightly acidic electrolyzed water (SAEW), fumaric acid (FA), and ultravioletC waterproof light-emitting diodes (UVC W-LED) for the control of *Staphylococcus aureus* and *Listeria monocytogenes* in fresh-cut fruits. Cherry tomato, grape, apple, and pineapple were inoculated with *S. aureus* and *L. monocytogenes* and then washed with 30 ppm SAEW containing 0.5% FA in a container equipped with two UVC W-LEDs. Behaviors of *S. aureus* and *L. monocytogenes* and quality properties of fresh-cut fruits were monitored after storage at 10 °C and 15 °C for 7 days. The most effective reductions of *S. aureus* (1.65 log CFU/g) and *L. monocytogenes* (2.63 log CFU/g) were observed in the group with the combined treatment of SAEW + FA and UVC W-LED. At 10 °C and 15 °C, populations of both pathogens in the combined treatment group were lower than those in a control. Combined treatment showed no negative effect on moisture retention in the fruit. Moreover, visual changes were less significant than in the control. These results demonstrate that the combined treatment can improve the microbial safety and the quality of fruits. If it is properly used in the sanitizing step of the fresh produce industry, a positive effect can be expected.

## 1. Introduction

Fresh produce is an important component of a healthy diet as it is a major source of fiber and micronutrients, including vitamins and functional compounds, such as polyphenolics, glucosinolates, and carotenoids [1]. In recent years, fresh-cut produce has gained great popularity among customers worldwide as healthy and convenient foods. The scale of the fresh-produce market in 2018 was estimated to be 808.9 billion won per year, which is expected to increase gradually in Korea [2]. Apples, pineapples, grapes, cherry tomatoes, and melons are popular fresh-cut fruits in Korea.

Foodborne diseases resulting from contaminated fresh produce have been reported globally [3]. In particular, fresh-cut fruit can be a high-risk food due to the chance of cross-contamination during the manufacturing process, such as peeling, slicing, dicing, and shredding. The lack of food safety management systems in the fresh-cut fruits industry has resulted in the outbreak of foodborne diseases [4,5]. The most common pathogenic bacteria isolated from fresh produce were *Escherichia coli*, *Salmonella*, *Listeria monocytogenes*, and *Staphylococcus aureus* [6,7]. Minimally processed fruits or fresh-cut fruits are often contaminated by *S. aureus* and *L. monocytogenes* that can survive and grow during transportation and the retail market [8,9]. Therefore, there is a need to effectively use inactivation techniques of foodborne pathogens before fresh-cut fruits reach consumers.

In general, washing with sanitizers such as sodium hypochlorite solution, chlorine dioxide, and ozone water has been used to reduce the microbial load on fresh-cut fruits [10,11]. However, previous studies [12,13] reported that excessive amounts of free chlorine could form with sodium hypochlorite washing, and accumulate disinfection by-products such as trihalomethan, so alternative methods are being investigated to overcome such problems.

Hurdle technology commonly refers to the application of a combined preservation method. It can be used as an alternative to the limited effectiveness of conventional disinfectant washing. It is also a potential technology that can reduce loss of quality while improving food safety [14]. Slightly acidic electrolyzed water (SAEW) is evaluated as an eco-friendly alternative disinfectant [13]. Fumaric acid (FA) has a strong antibacterial effect among organic acids [15]. Combining FA and SAEW can effectively inactivate *E. coli* O157:H7, *L. monocytogenes*, *Salmonella* spp. in various fresh produce, such as lettuce, sprouts, spinach, and apples [15,16]. These novel technologies may produce great synergistic antimicrobial activity compared to a single treatment [17].

Ultraviolet (UV) radiation near 260 nm is part of the UVC radiation wavelength band that can destroy microbial DNAs. It is considered the most effective germicidal region in the UV spectrum [18]. As a novel UV source, UV-LEDs have become potential alternatives to conventional UV mercury lamps due to their advantages, such as a long lifetime, eco-friendliness (no mercury), diversity in wavelengths, and no warm-up time being needed [19,20]. In particular, UVC waterproof LED has the advantage of being differentiated from conventional UV treatment, as it can simultaneously perform washing and irradiation. Since the product and washing water are sterilized at the same time during processing, cross-contamination caused by washing water can be prevented using UVC waterproof LED. There has been no research on UV waterproof LED applied in the food industry. Therefore, the objectives of this study were to evaluate the antimicrobial effects of hurdle technologies (SAEW + FA and UVC W-LED) against *L. monocytogenes* and *S. aureus* on fresh-cut fruits. In addition, the possibility of extending the shelf life of treating fresh-cut fruits with these hurdle technologies was investigated.

## 2. Materials and Methods

### 2.1. Strain Preparation

*Listeria monocytogenes* strains (ATCC 15313, ATCC 19111) were purchased from the Korean Culture Center of Microorganisms (KCCM). A strain of *L. monocytogenes* isolated from the smoked salmon in the online market was also used. Enterotoxin A-producing *Staphylococcus aureus* (SEA; ATCC13565) was purchased from the American Type Culture Collection (ATCC). Enterotoxin G- and I-producing *S. aureus* and non-enterotoxin-producing *S. aureus* were isolated from red cabbage and pineapple, respectively. They were used as a cocktail strain for inoculation in this study. Each stock culture of *L. monocytogenes* and *S. aureus* was maintained in tryptic soy broth (TSB, MBcell, Seoul, Korea) containing 0.6% yeast and TSB, respectively. All strains were stored at −80 °C in TSB containing 20% glycerol. *L. monocytogenes* and *S. aureus* (10 µL each) were inoculated into 10 mL of sterilized TSB containing 0.6% yeast extract and TSB, respectively. These inoculated bacteria were cultured at 36 °C for 24 h with shaking (140 rpm) using a rotary shaker (VS-8480SP, Vision, Daejeon, Korea). Equal quantities of each pre-cultured strain were mixed in a 50 mL conical tube (SPL life Sciences, Pocheon, Korea) to make culture cocktails for experiments.

### 2.2. Preparation of Sample and Inoculation

Fresh apples, pineapples, grapes, and cherry tomatoes were purchased from offline markets (Dongdaemun-gu, Seoul) in Korea and kept at 4 °C until used. After removing stems, approximately the same size and weight of cherry tomatoes and grapes (10 g) were used, and 20 µL of cocktail strains were inoculated onto the stem scar. Peeled pineapples and apples were aseptically cut into 10 g each (thickness: pineapple, 1.5–2 cm, apple, 2–3 cm) and inoculated with 100 µL of cocktail strains. These inoculated samples were air-dried for approximately 30 min on a clean bench to allow bacteria to attach to surfaces of fruits.

### 2.3. Sanitizing Solution with SAEW and FA

The SAEW was produced using a SAEW generator that consisted of a non-membrane electrolytic chamber (BC-120, Cosmic Round Korea Co., Seongnam, Korea). The pH and available chlorine concentrations (ACC) of SAEW were measured using a pH meter (Orion-star pH-Benchtop, Thermo, Waltham, MA, USA) and chlorine test papers (Toyo Roshi Kaisha, Ltd., Tokyo, Japan), respectively. SAEW had a pH of 5.5 and an ACC of 30 ppm. Fumaric acid (FA, 99.0%, Sigma-Aldrich, St. Louis, MO, USA) was directly diluted with SAEW using a magnetic stirrer to obtain a final concentration of 0.5% (*w*/*v*).

### 2.4. UVC Waterproof Lights Emitting Diodes (UVC W-LED)

UV treatments were performed in a stainless-steel case (32.5 cm × 17.5 cm × 15 cm) equipped with two UVC W-LED (275 nm) modules (BlueLumi Co., Ltd., Gyeonggi-do, Korea), which were placed on each side of a stainless-steel case.

### 2.5. Decontamination with SAEW, FA, and UVC W-LED

Proposed hurdle treatments of SAEW, FA, and UVC W-LED are shown in Table 1. Sodium hypochlorite (NaClO; Hanson Hygiene Co., Korea) at 100 ppm as control was prepared by diluting 4% NaClO solution using distilled water.

For a single treatment, samples were washed only with 100 ppm NaClO or 30 ppm SAEW. For combined treatment, samples were washed with 30 ppm SAEW containing 0.5% FA for 3 min. For the combined treatment with SAEW + FA and UVC W-LED, samples were placed in the stainless container, which was equipped with two UVC W-LEDs and washed with the mixed solution (30 ppm SAEW and 0.5% FA). All treatments were performed at room temperature.

### 2.6. Microbiological Analysis

To investigate the reduction effect of each treatment and its impact on the growth of pathogenic bacteria, the number of viable cells was measured using a spread plate culture method. After disinfection treatment, 10 g of samples were homogenized with sterile 0.1% peptone water (BD, Sparks, NV, USA) using a stomacher (Interscience, St Nom la Bretêche, France) for 120 s. Subsequent decimal dilutions in peptone water were then plated onto selective media: Baird-Parker agar (BPA, MB cell, Seoul, Korea) and PALCAM agar (Oxoid, Hampshire, England) were used for enumeration of *S. aureus* and *L. monocytogenes*, respectively. As a nonselective media, tryptic soy agar (TSA, MBcell, Seoul, Korea) containing 0.6% yeast extract and TSA were used for *L. monocytogenes* and *S. aureus*, respectively. Both selective and nonselective media were incubated at 37 °C for 24–48 h. Colonies on the plate were then counted using a colony counter (Scan 1200, Interscience, St Nom la Bretêche, France). Microbial count was expressed as log CFU/g.

### 2.7. Quality Analysis of Treated Fresh-Cut Fruits during Storage at 10 °C and 15 °C

To analyze effects of hurdle technologies on qualities of treated samples, the overall visual appearance, color, moisture loss of fresh-cut fruits, and survival of pathogens on fresh-cut fruit were investigated during storage at 10 °C and 15 °C for 7 days. Fresh-cut fruit was washed with 30 ppm SAEW containing 0.5% FA with and without UVC W-LED for 3 min. After treatment, samples were packaged using PET containers (Modenpack, Seoul, Korea) and stored at 10 °C (storage temperature) or 15 °C (abused temperature).

#### 2.7.1. Changes in Populations of *S. aureus* and *L. monocytogenes* on Fresh-Cut Fruit after Combined Treatment

The growth and survival of *S. aureus* and *L. monocytogenes* on treated samples were investigated for 7 days after storage at 10 °C and 15 °C. Samples without any extra wash with a sanitizer were used as controls.

#### 2.7.2. Visual Change and Measurements of the Color

The overall visual defect was used as a tool to evaluate the change in fruit quality. Treated samples and controls were regularly photographed on a white plate every day during storage at 10 °C and 15 °C. The color of fresh-cut fruit was analyzed in triplicate using a colorimeter (Minolta CR-400, Osaka, Japan) every day for 7 days at room temperature. Color values of samples were expressed as L* (lightness), a* (red–green chromaticity), and b* (yellow–blue chromaticity) during each measurement.

#### 2.7.3. Moisture Loss

Moisture loss (%) of treated fresh-cut fruit during storage was calculated by subtracting the final weight of the fruit from its initial weight, divided by the initial weight, and then multiplied by 100 [21]. Each sample was weighed to within ±0.001 g using a sensitive electronic balance (WBA-320, DAIHAN Scientific Co., Ltd., Wonju, Korea):

(%) Moisture loss = (initial mass − final mass)/initial mass × 100.(1)

### 2.8. Statistical Analysis

The experiment was repeated twice with three replicates per experiment. The significance of differences among or between samples was determined by one-way analysis of variance (ANOVA) followed by Duncan’s multiple range tests or *t*-test. Data are expressed as mean ± standard deviation (SD). Significance was considered when *p <* 0.05. All statistical analyses were performed using the statistical software program SAS release 9.4 (SAS Institute, Inc., Cary, North Carolina, USA).

## 3. Results and Discussion

### 3.1. The Sanitizing Effect with Combined SAEW and FA on Microbiological Hazards in Fresh-Cut Fruit

**Figure 1:**
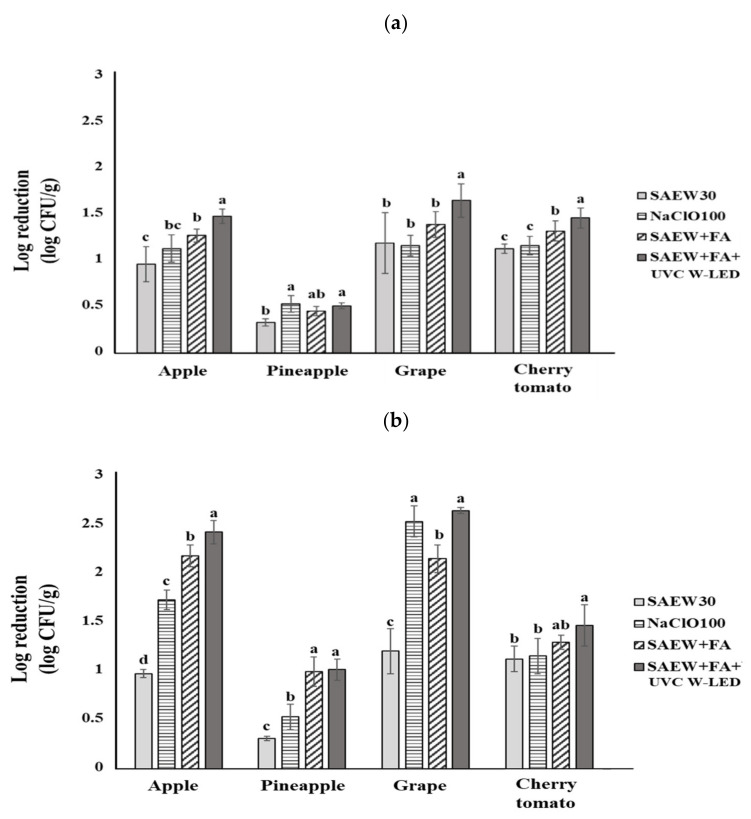
Reduction effect of (**a**) *S. aureus* and (**b**) *L. monocytogenes* after disinfection treatments in various fruits. NaClO, sodium hypochlorite solution (control, 100 ppm); SAEW, slightly acidic electrolyzed water (30 ppm); FA: fumaric acid (0.5%); UVC W-LED: ultravioletC waterproof light-emitting diodes (275 nm). ^a–d^ values within each fruit represent differences by using Duncan’s multiple-range test at *p* < 0.05.

Results of decontaminating *S. aureus* and *L. monocytogenes* on apple, pineapple, grape, and cherry tomato by sanitizing with combined SAEW and FA are summarized in Figure 1. After 3 min of dipping treatment with 30 ppm SAEW, reductions of populations of *S. aureus* ranged from 0.33 to 1.19 log CFU/g. Under identical conditions, *L. monocytogenes* populations showed reductions of 0.31–1.20 log CFU/g. The combination of SAEW + FA (0.5%) resulted in higher bacterial reductions than a SAEW single treatment for fresh-cut fruit. The highest reduction of *S. aureus* was 1.39 log CFU/g for grapes, followed by that for cherry tomatoes (1.32 log CFU/g), apples (1.27 log CFU/g), and pineapples (0.45 log CFU/g). Populations of *L. monocytogenes* on apples, grapes, cherry tomatoes, and pineapples were reduced by 2.17, 2.14, 1.29, and 0.99 log CFU/g, respectively. The least reduction effects of both pathogens were observed for pineapples in this work.

Washing with SAEW has been reported as an effective method for the reduction of pathogens [14,22,23]. Ding et al. [24] have revealed that treatment of cherry tomatoes with SAEW can result in total aerobic bacteria reduction of 1.45 log CFU/g and 1.10 log CFU/g reduction for yeasts and mold. After inoculated bell peppers were treated for 3 min with SAEW alone at room temperature [23], reductions of 1.25 log CFU/g for *L. monocytogenes* and 1.19 log CFU/g for *S.* Typhimurium were reported, similar to results of the present study.

Several studies have reported that the combination of SAEW with chemical/physical treatments can result in a higher reduction effect of microbes than a single SAEW treatment [16,25,26]. SAEW + FA treatments significantly (*p* < 0.05) reduced counts of *E. coli* O157:H7, *L. monocytogenes*, and *Salmonella* spp. on lettuce compared to SAEW single treatments [16]. Guo et al. [25] revealed that US and SAEW treatment shows a synergistic sterilizing effect by significantly reducing the number and particle size (762 nm) of *E. coli* compared to a single treatment, in which the average particle size of *E. coli* is 1436 nm. According to Hussain et al. [26], treatment with SAEW (80 ppm) and mild heat (60 °C) for 10 min can result in additional reductions of 0.76 log CFU/mL for *Bacillus cereus* spores compared to SAEW single treatments.

### 3.2. The Sanitizing Effect of Combined SAEW and FA with UVC W-LED

Effects of various combined treatments on microorganism inactivation have been reported in produce. However, little information is available for fresh-cut fruits. In this study, inactivation efficacies of combined SAEW + FA and UVC W-LED treatments on *S. aureus* and *L. monocytogenes* in various fresh-cut fruits were evaluated. Results are shown in Figure 1.

Overall, the combination treatment of SAEW + FA and UVC W-LED resulted in a greater disinfection effect for *S. aureus* and *L. monocytogenes* (0.51 to 2.63 log CFU/g reduction) in fresh-cut fruits than SAEW single treatment or combined treatment of SAEW containing 0.5% FA (0.31 to 2.17 log CFU/g reduction). In all experimental groups, the combined treatment of SAEW + FA and UVC W-LED was observed to have a greater reduction effect than 100 ppm NaClO, a sanitizer commonly used in the produce industry, except for *S. aureus* inoculated to pineapples. When the combined treatment was applied, populations of *S. aureus* on grapes, apples, cherry tomatoes, and pineapples were reduced by 1.65, 1.48, 1.46, and 0.51 log CFU/g, respectively. Reductions for counts of *L. monocytogenes* on grapes, apples, cherry tomatoes, and pineapples were 2.63, 2.41, 1.46, and 1.01 log CFU/g, respectively. The highest reductions of both pathogens were observed for grapes, followed by apples, cherry tomatoes, and pineapples. The lowest reduction effects on both *S. aureus* and *L. monocytogenes* were observed for pineapples. This might be because the surface of pineapple is uneven and rough. Thus, reduction effects on *S. aureus* and *L. monocytogenes* were less than those on other samples. Previous studies have shown that fruit surface roughness and smoothness can influence the effectiveness of reducing bacterial populations. When fruit surface roughness increases, the efficacy of microbial inactivation decreases [24,27]. Moreover, the difference in the reduction effect among samples or bacteria shown in this experiment could be due to different sensitivities of microorganisms to each hurdle technique. Many factors, including bacterial species, surface topography, and transmittance of food, wavelength, and physical arrangement of the UV source, can affect the reduction effect on microorganisms [28,29].

Previous studies have been conducted to reduce pathogenic bacteria in mixed beverages, apple juice, and sliced cheese using UV-LED treatment [20,30,31]. Combining UV-LED and other treatments has been reported in various studies [32,33,34]. UV-LED (280 nm, for 5 min) combined with SAEW (20 ppm, for 3 min) could reduce *E. coli* O157:H7 by 1.41 log CFU/g and *Salmonella* by 1.68 log CFU/g on coriander [32]. The population of *Salmonella* is decreased by 2.97 log CFU/g in lettuce after a sequential treatment with 80 ppm SAEW for 2 min and UVC-LED (100 μW/cm^2^) exposure for 30 min [33]. Recently, Lee et al. [34] have revealed that inactivation of pathogenic *E. coli* and *S. aureus* on fresh-cut vegetables can be achieved in the range of 0.97 to 2.17 log CFU/g with SAEW and ultrasounds, followed by UV-LED (4.14 mJ/cm^2^, 275 nm) treatment before packaging.

There is no research that applies UVC W-LED in the food industry. This study was the first trial of UVC W-LED for fresh-cut fruit. It proved that UVC W-LED combined with SAEW and FA was effective in reducing food-borne pathogens on fresh-cut fruit. These results indicate that the use of a combined treatment (SAEW + FA and UVC W-LED) can be applied as a novel hurdle technology in the fresh-cut fruits industry. The fundamental principle of projecting UV light onto submerged surfaces to prevent marine biofouling has been also successfully verified by Ryan et al. [35].

### 3.3. Effects of Treatments of SAEW, FA, and UVC W-LED on Behaviors of S. aureus and L. monocytogenes on Fresh-Cut Fruits after Storage at 10 °C and 15 °C

**Figure 2:**
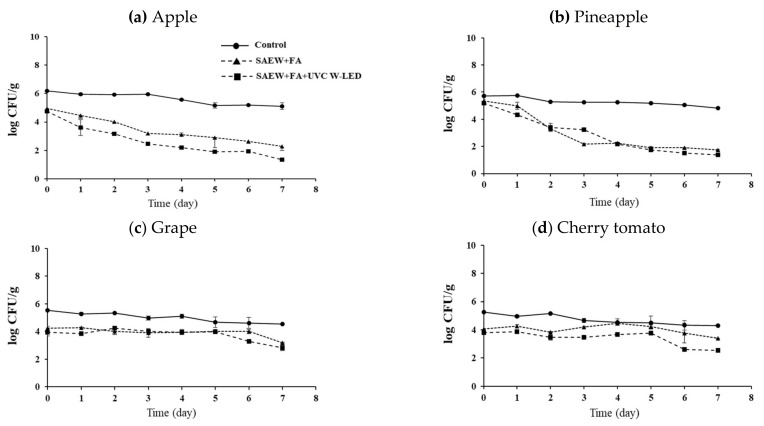
Change of *S.aureus* population of (**a**) apple, (**b**) pineapple, (**c**) grape and (**d**) cherry tomato with disinfection treatments during storage at 10 °C. Control, no extra wash with a sanitizer; SAEW, slightly acidic electrolyzed water; FA, fumaric acid; UVC W-LED, ultravioletC waterproof light-emitting diodes. Control (●), SAEW + FA (▲), SAEW + FA + UVC W-LED (■).

**Figure 3:**
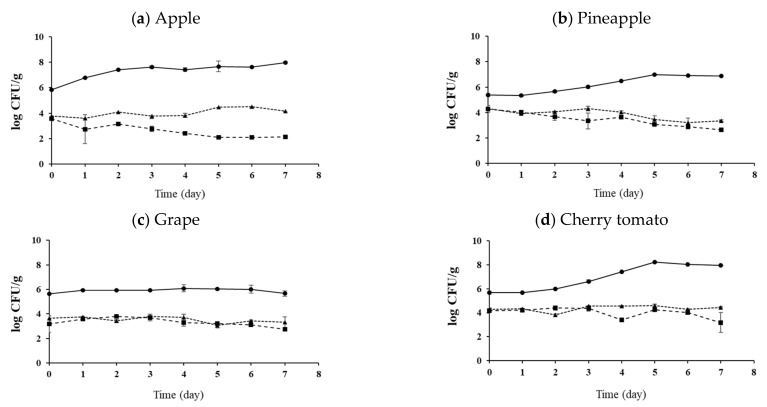
Change of *L. monocytogenes* population of (**a**) apple, (**b**) pineapple, (**c**) grape and (**d**) cherry tomato with disinfection treatments during storage at 10 °C. Control, no extra wash with a sanitizer; SAEW, slightly acidic electrolyzed water; FA, fumaric acid; UVC W-LED, ultravioletC waterproof light-emitting diodes. Control (●), SAEW + FA (▲), SAEW + FA + UVC W-LED (■).

**Figure 4:**
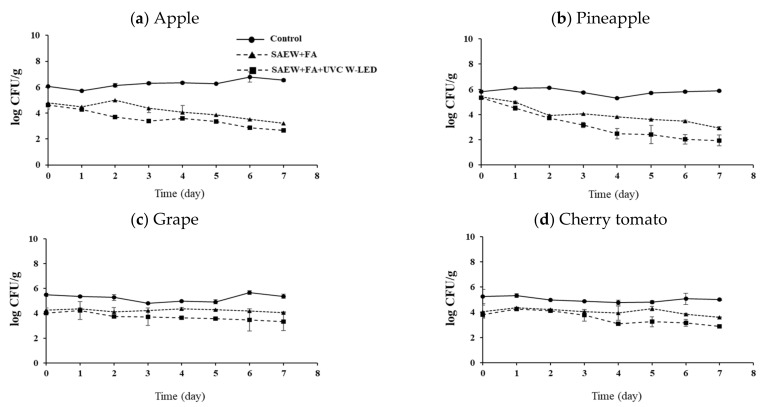
Change of *S.aureus* population of (**a**) apple, (**b**) pineapple, (**c**) grape and (**d**) cherry tomato with disinfection treatments during storage at 15 °C. Control, no extra wash with a sanitizer; SAEW, slightly acidic electrolyzed water; FA, fumaric acid; UVC W-LED, ultravioletC waterproof light-emitting diodes. Control (●), SAEW + FA (▲), SAEW + FA + UVC W-LED (■).

**Figure 5:**
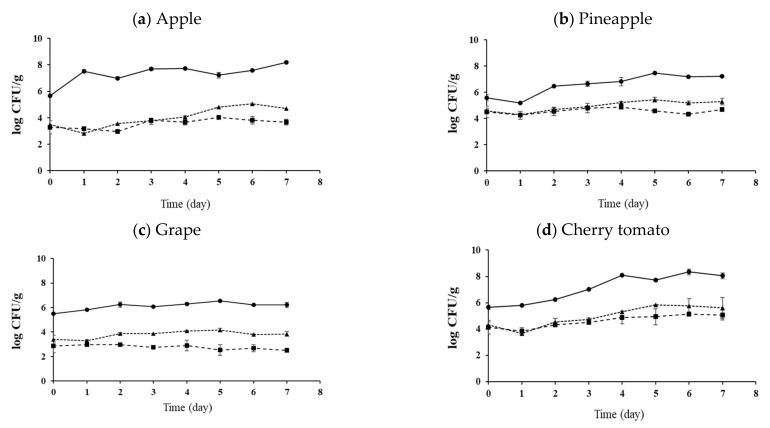
Change of *L. monocytogenes* population of (**a**) apple, (**b**) pineapple, (**c**) grape and (**d**) cherry tomato with disinfection treatments during storage at 15 °C. Control, no extra wash with a sanitizer; SAEW, slightly acidic electrolyzed water; FA, fumaric acid; UVC- W LED, ultravioletC waterproof light-emitting diodes. Control (●), SAEW + FA (▲), SAEW + FA + UVC-W LED (■).

Fresh-cut fruits were treated with SAEW + FA or SAEW + FA and UVC W-LED. The experimental group without an extra wash with a sanitizer was used as a control group for comparison. Effects of hurdle treatments on the changes of pathogen populations on apples, pineapples, grapes, and cherry tomatoes during storage at 10 °C and 15 °C for 7 days are shown in Figure 2, Figure 3, Figure 4 and Figure 5, respectively. As described in the previous section, initial numbers of *S. aureus* and *L. monocytogenes* on fresh-cut fruits were more reduced with combined treatments than with the control.

At 10 °C, the SAEW + FA and UVC W-LED reduced populations of both pathogens to the lowest level for all fresh-cut fruits after 7 days of storage (Figure 2 and Figure 3). The population of *S. aureus* was decreased in all treatment groups. The greatest reduction was observed in the SAEW + FA and UVC W-LED treated group. In the case of apples (Figure 2a), the control group showed reduction by 1 log CFU/g during 7 days of storage, while the SAEW + FA treatment group showed a decrease of 2.6 log CFU/g and the SAEW + FA and UVC W-LED treatment group showed a decrease of 3.4 log CFU/g. Similar results were also observed for pineapples (Figure 2b). Less reductions (about 1 log CFU/g) were observed for cherry tomatoes and grapes than for apples and pineapple during 7 days of storage. The population of *L. monocytogenes* decreased or was maintained in the SAEW + FA and UVC W-LED treatment group during storage, while the population of *L. monocytogenes* increased continuously in the control group except for grapes (Figure 3c).

At 15 °C, the population of *S. aureus* decreased or maintained in samples treated with SAEW + FA or SAEW + FA and UVC W-LED compared to that in the control group (no extra wash with sanitizer) after 7 days of storage (Figure 4). The lowest counts of *S. aureus* were observed for apples and pineapples (Figure 4a,b). The population of *L. monocytogenes* continued to increase rapidly in the control group, while they were maintained or increased slowly in samples treated with SAEW + FA and UVC W-LED (Figure 5). Changes in behaviors of bacteria during storage after disinfection have been reported previously [32,34,36]. Total aerobic bacteria (TAB) on untreated mandarin increased faster than those on FA + SAEW treated mandarin. After storage at 4 °C for 14 days, the final population of TAB was observed at significantly lower levels in treated mandarins than in untreated samples (*p* < 0.05) [36]. According to Jiang et al. [32], the total number of *Salmonella* and *E. coli* in coriander treated with SAEW (60 ppm, for 5 min) and UV-LED (240 μW/cm^2^, for 30 min) and stored at 4 °C for 6 days showed a slow growth trend. Surface microorganisms of the treatment group were also always lower than those of the control group. Lee et al. [34] have also confirmed that populations of *S. aureus* and *E. coli* O157:H7 are decreased in fresh-cut vegetables treated with SAEW, US, and UV-LED during storage at 5 °C and 15 °C. Growths of *S. aureus* and *E. coli* O157:H7 on carrots and celeries were inhibited during storage at 15 °C after they were treated with SAEW, US, and UVC LED.

In this study, greater growth controls for pathogens were observed for apples and pineapples than for grapes and cherry tomatoes during storage. Because UV has a limited penetration depth to fruit tissues, it has an antibacterial effect through surface sterilization [37]. Since we inoculated pathogens to stem scars of grapes and cherry tomatoes, pathogens could enter the inside of the fruit pericarp through the stem scar. Thus, the antibacterial action with UVC W-LED against bacteria might be insufficient. Because grapes and cherry tomatoes are covered with pericarps, treatments seem to have less effects on bacteria reduction of these samples during storage compared to fresh-cut apples and pineapples.

Our results clearly showed that the combination treatment with SAEW + FA and UVC W-LED could extend the shelf-life by improving the microbial safety of fresh-cut fruits during storage. However, the bactericidal efficacy depended on the type of fruit. These findings indicate that UVC W-LED is a promising decontamination hurdle technology that can be applied at the sanitizing step in the fresh produce industry.

### 3.4. Quality Analysis of Treated Fresh-Cut Fruits during Storage at 10 and 15 °C 

#### 3.4.1. Moisture Loss

**Figure 6:**
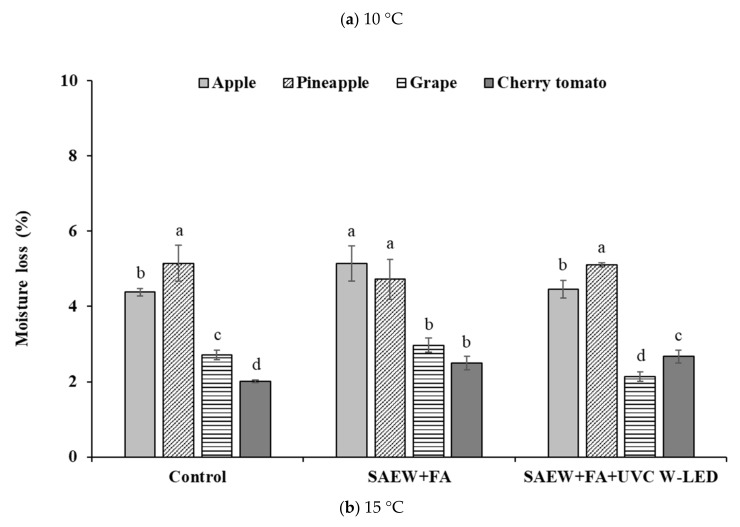

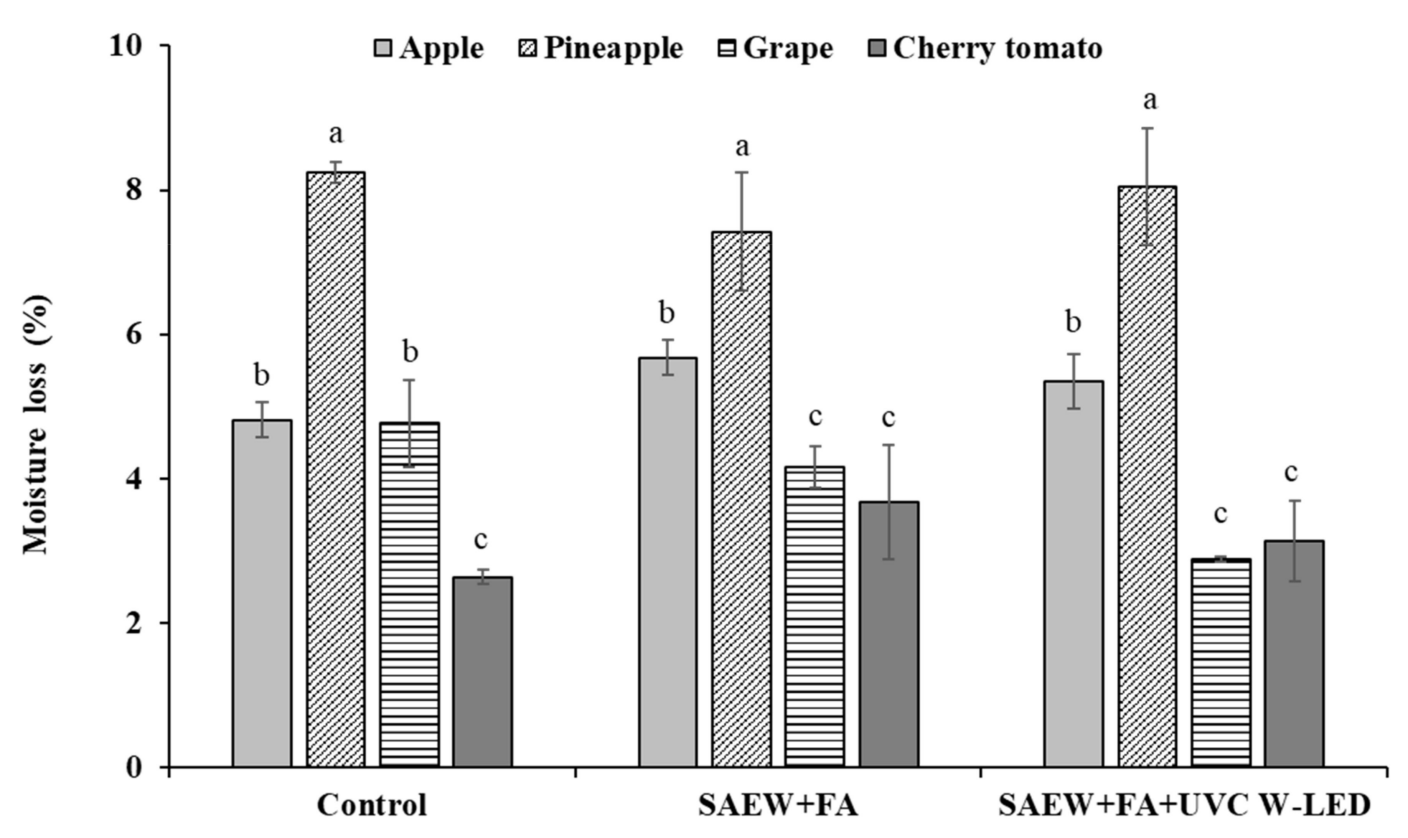
Effectiveness of disinfection treatments on the moisture loss in various fresh-cut fruits stored after 7 days at (**a**) 10 °C and (**b**) 15 °C. Control, no extra wash with a sanitizer; SAEW, slightly acidic electrolyzed water; FA, fumaric acid; UVC W- LED, ultravioletC waterproof light-emitting diodes. ^a–d^ values within each treatment represent different by Duncan’s multiple range test at *p* < 0.05.

Moisture loss has a negative effect on the product quality of certain fruits and vegetables as it can lead to bad texture, change of shape, and poor color, resulting in the loss of profitability of fresh produce [38]. In this study, relative moisture losses of fruits from effects of different treatments during storage for 7 days at 10 °C and 15 °C were determined. Results are shown in Figure 6. Moisture losses of various fruits increased during storage at 10 °C (Figure 6a) and 15 °C (Figure 6b) in all groups. After storage at 10 °C for 7 days, the percentages of moisture loss ranged from 4.38 to 5.14% in apples, 2.14 to 2.97% in grapes, 4.72 to 5.15% in pineapples, and 2.01 to 2.67% in cherry tomatoes. At 15 °C, the maximum moisture losses were 8.24, 5.68, 4.77, and 3.67% in pineapples, apples, grapes, and cherry tomatoes, respectively. Although moisture losses of fresh-cut fruits increased as the storage temperature increased from 10 °C to 15 °C, in general, no significant difference in weight loss was observed among treatment groups. Moisture loss was significantly less in grapes treated with SAEW + FA and UVC W-LED compared to those after washing treatment without UVC W-LED or in the control (no extra wash with a sanitizer).

These results indicated that SAEW + FA or SAEW + FA and UVC W-LED treatment caused no negative effect on moisture loss of fruits compared to the control, suggesting that the treatment used in this study could be applied to the fresh-cut fruit industry. Results of this study were similar to those of previous studies [37,38]. When strawberries were stored for 8 days at 6 °C after pulsed-light treatment (2.4–23.9 J/cm^2^), there were no significant differences in moisture loss between treated and untreated groups [39]. According to Sripong et al. [40], there was no significant difference in moisture loss between mangoes treated with hot water (55 °C, 5 min) + UVC (6.16 kJ m^−2^) and those in the control group.

In this research, the greatest moisture was observed for pineapples at both storage temperatures, while cherry tomatoes showed less moisture loss. Cherry tomatoes had less moisture loss than pineapples, possibly because the pulp was not directly exposed to the outside due to the skin surrounding the juicy pulp.

#### 3.4.2. Visual and Color Changes of Treated Fresh-Cut Fruits during Storage at 10 °C and 15 °C

**Figure 7:**
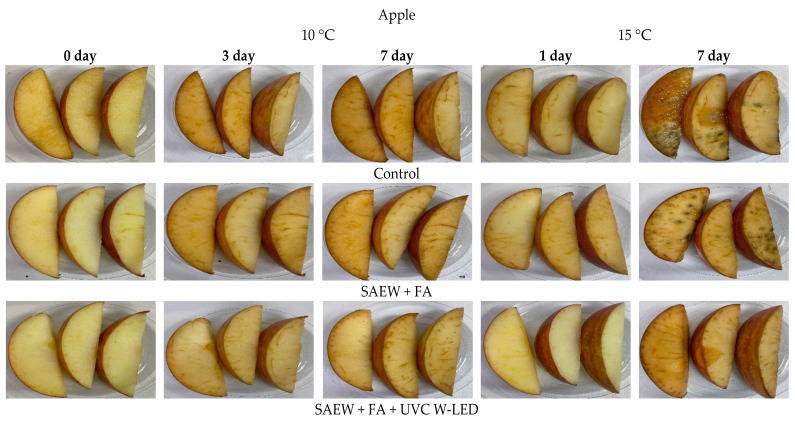
Changes in the overall visual quality of apples with disinfection treatments after storage for 7 days at 10, 15 °C. Control, no extra wash with a sanitizer; SAEW, slightly acidic electrolyzed water; FA, fumaric acid; UVC W-LED, ultravioletC waterproof light-emitting diodes.

**Figure 8:**
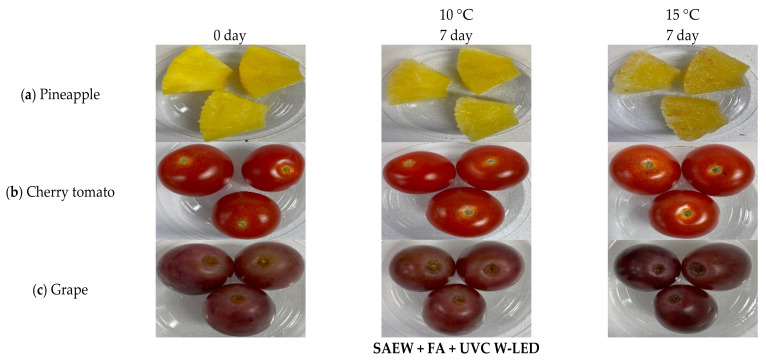
Changes in the overall visual quality of pineapples, cherry tomatoes, and grapes treated with a combination of SAEW + FA and UVC W-LED after storage for 7 days at 10, 15 °C. SAEW, slightly acidic electrolyzed water; FA, fumaric acid; UVC W-LED, ultravioletC waterproof light-emitting diodes.

Appearance changes of treated fresh-cut fruit stored at 10 °C and 15 °C for 7 days are shown in Figure 7 and Figure 8. Overall, visual changes of fresh-cut fruit were less significant in treated groups than in the control during 7 days of storage. In particular, remarkable appearance changes of control were identified compared to those of apples treated with SAEW + FA and UVC-W LED during storage at both temperatures (Figure 7).

At 15 °C, the control group of apples began to show browning after one day of storage. However, visual changes of treated apples with SAEW + FA and UV-C W LED were relatively less progressed during the same period. In this work, the browning of fresh-cut apples was not completely controlled by the combined treatment. According to previous research [41], ascorbic acid showed higher inhibition effect on browning than other solutions. Therefore, in terms of anti-browning, ascorbic acid is thought to be more effective than FA used in this experiment. Browning is less of a concern with pineapples, cherry tomatoes, and grapes. The overall appearance of pineapples was well-maintained in the treatment group than in the control. Mold was observed in the control group of tomatoes (data not shown).

The visual appearance of color is known as the main factor influencing consumers’ choices. Fresh produce undergoes complicated chemical and biochemical changes during post-harvest handling, resulting in slight color changes [42]. In this study, color parameters of fresh-cut fruits were consistent with the appearance quality (Table 2 and Table 3). Generally, L*, a*, and b* values of samples treated with combined SAEW + FA and UVC W-LED were significantly (*p* < 0.05) different from those of controls. These results were most noticeable for apples stored at 10 °C. L* values of treated apples were significantly higher those of controls, indicating that treatment prevented the browning of apples. Furthermore, pineapples treated with SAEW + FA and UVC W-LED showed significantly higher L* values than the control group of pineapples stored at 15 °C.

The main problem with fresh-cut fruit is related to color changes, which significantly limits their shelf life. Browning is caused by the interaction of polyphenol oxidase with phenol released during minimal processing [43,44]. Conversely, the antioxidant activity of fruits is caused by phenolic compounds. Previous studies have shown that UVC treatment can increase total polyphenols in mangoes and pineapples and that the antioxidant activity is significantly increased compared to the control group [44]. In addition, UVC irradiation can inactivate polyphenol oxidase activity, preventing further loss of polyphenols [45]. With the same mechanism, UV-LED treatment has been proven to be effective in inactivating the polyphenol oxidase of apple juice [20]. These results indicate that the combined treatment of SAEW + FA and UVC W-LED can be an effective method to extend the shelf life of fresh-cut fruits without adversely affecting their quality parameters.

## 4. Conclusions

This study evaluated antibacterial effects of hurdle technologies using 30 ppm SAEW containing 0.5% FA and UVC waterproof LED and the possibility of extending the shelf life of treated fresh-cut fruits with these hurdle technologies. The combination treatment of SAEW + FA and UVC W-LED resulted in a greater disinfection effect on *S. aureus* and *L. monocytogenes* in grapes, cherry tomatoes, fresh-cut apples, and pineapples compared to other treatments. The least reduction effect on both pathogens was observed for pineapples.

At 10 °C and 15 °C, populations of *S. aureus* and *L. monocytogenes* with combined treatment were observed at a lower level than the control group. Overall, no significant difference in moisture loss was observed among treatment groups. However, visual changes of fresh-cut fruits were less significant in treated groups than in the control group during 7 days of storage. They were prominent for fresh-cut apples and pineapples. Results of this study indicate that UVC W-LED is a promising decontamination hurdle technology that can be applied at the sanitizing step in the fresh produce industry. It can be an effective method to extend the shelf life of fresh-cut fruits without adversely affecting their quality parameters.

## Figures and Tables

**Table 1:** Application conditions of each treatment for fresh-cut fruits.

Treatments	Washing Time
100 ppm NaClO	5 min
30 ppm SAEW	3 min
30 ppm SAEW + 0.5% FA	
30 ppm SAEW + 0.5% FA + UVC W-LED	

NaClO, sodium hypochlorite solution (control, 100 ppm); SAEW, slightly acidic electrolyzed water (30 ppm); FA: fumaric acid (0.5%); UVC W-LED; ultravioletC waterproof light-emitting diodes (275 nm).

**Table 2:** Effectiveness of disinfection treatments on color parameters in apple and pineapple after storage for 7 days at 10 °C.

Treatment	Storge Time	Apple ^1^			Pineapple		
		L*	a*	b*	L*	a*	b*
Control	0 day	^B^ 64.11 ± 0.68	^C^ −0.68 ± 0.54	^B^ 22.31 ± 0.23	^A^ 64.09 ± 1.75	^D^ −5.91 ± 0.30	^A^ 27.52 ± 0.30
	7 day	^E^ 52.80 ± 1.22	^A^ 1.84 ± 0.38	^A^ 24.97 ± 0.34	^B^ 44.98 ± 1.32	^A^ −0.68 ± 0.26	^D^ 13.99 ± 0.63
SAEW + FA	0 day	^A^ 70.95 ± 1.81	^C^ −1.36 ± 0.48	^C^ 20.45 ± 0.25	^A^ 63.99 ± 0.87	^D^ −5.50 ± 0.20	^B^ 25.84 ± 0.48
	7 day	^D^ 57.99 ± 1.23	^A^ 2.57 ± 0.35	^B^ 22.01 ± 0.27	^B^ 47.90 ± 0.32	^B^ −1.89 ± 0.76	^C,D^ 14.31 ± 0.93
SAEW + FA +UVC W-LED	0 day	^A^ 72.49 ± 0.71	^D^ −2.85 ± 0.61	^C^ 20.14 ± 0.58	^A^ 63.38 ± 2.41	^D^ −6.02 ± 0.09	^B^ 26.30 ± 0.12
	7 day	^C^ 60.89 ± 1.21	^B^ 0.85 ± 0.07	^B^ 21.91 ± 0.20	^B^ 48.44 ± 1.75	^C^ −3.97 ± 0.06	^C^ 15.76 ± 0.28

^1^ Means ± standard deviation (*n* = 3). Control, no extra wash with a sanitizer; SAEW, slightly acidic electrolyzed water; FA, fumaric acid; UVC W-LED, ultravioletC waterproof light-emitting diode. ^A–E^ Means values in the same column with different letters are significantly different (*p* < 0.05).

**Table 3:** Effectiveness of disinfection treatments on color parameters in apple and pineapple after storage for 7 days at 15 °C.

Treatment	Storge Time	Apple ^1^			Pineapple		
		L*	a*	b*	L*	a*	b*
Control	0 day	^B^ 63.64 ± 2.46	^B^ −0.38 ± 0.10	^C,D^ 21.94 ± 0.51	^A,B^ 62.77 ± 1.86	^C^ −5.65 ± 0.17	^A^ 26.79 ± 0.14
	7 day	^C^ 54.98 ± 3.80	^A^ 2.85 ± 0.96	^A^ 26.23 ± 0.46	^D^ 40.65 ± 0.25	^A^ −0.11 ± 0.80	^B^ 11.16 ± 1.16
SAEW + FA	0 day	^A,B^ 68.08 ± 1.67	^B^ −0.03 ± 0.10	^D,E^ 21.24 ± 0.49	^A^ 64.11 ± 1.14	^C^ −5.77 ± 0.07	^A^ 25.63 ± 0.90
	7 day	^C^ 54.59 ± 0.74	^A^ 2.35 ± 0.56	^B^ 24.47 ± 0.23	^D^ 42.98 ± 0.81	^A^ −0.97 ± 0.25	^B^ 12.20 ± 0.47
SAEW + FA +UVC W-LED	0 day	^A^ 70.85 ± 1.62	^C^ −2.48 ± 0.20	^E^ 20.60 ± 0.28	^B^ 61.32 ± 1.67	^C^ −5.18 ± 0.14	^A^ 25.45 ± 0.69
	7 day	^C^ 58.25 ± 2.75	^A^ 1.83 ± 0.26	^C^ 22.48 ± 0.28	^C^ 46.31 ± 0.31	^B^ -2.29 ± 0.60	^B^ 12.25 ± 0.23

^1^ Means ± standard deviation (*n* = 3). Control, no extra wash with a sanitizer; SAEW, slightly acidic electrolyzed water; FA, fumaric acid; UVC W-LED, ultravioletC waterproof light-emitting diode. ^A^^–E^ Means values in the same column with different letters are significantly different (*p* < 0.05).

## Data Availability

We did not report any additional data for this study.
